# Unmet healthcare needs in adults with childhood-onset neurodisabilities: a protocol for a systematic review

**DOI:** 10.12688/hrbopenres.13309.1

**Published:** 2021-09-28

**Authors:** Elaine Meehan, Aoife L. Gallagher, Jennifer Ryan, Claire Kerr, Rory O' Sullivan, Rose Galvin, Manjula Manikandan, Andrew Wormald, Katie Robinson

**Affiliations:** 1 Ageing Research Centre, Health Research Institute, University of Limerick, Limerick, Ireland; 2 School of Allied Health, University of Limerick, Limerick, Ireland; 3 Health Implementation Science and Technology Research Cluster, Health Research Institute, University of Limerick, Limerick, Ireland; 4 Division of Population Health Sciences, Royal College of Surgeons in Ireland, Dublin, Ireland; 5 School of Nursing and Midwifery, Queen's University Belfast, Belfast, UK; 6 Central Remedial Clinic, Dublin, Ireland

**Keywords:** Neurodisability, developmental disabilities, intellectual disability, cerebral palsy, ageing, unmet healthcare needs, healthcare access, health services research.

## Abstract

Background

Many adults with childhood-onset neurodisabilities, such as those with intellectual disability or cerebral palsy, report difficulties accessing the healthcare that they require when they are no longer eligible for paediatric services. Compared to the general population, this population is at greater risk of developing many ageing-related diseases and has higher rates of preventable deaths and premature mortality. Addressing unmet healthcare needs is essential to ensuring equitable access in a quality healthcare system. The aim of this systematic review is to synthesise the current available evidence related to unmet healthcare needs in adults with a range of childhood-onset neurodisabilities.

Methods

A systematic review of quantitative research studies of adults with a range of diagnoses that fall under the neurodisability umbrella and outcomes related to unmet healthcare needs will be undertaken. The Conducting Systematic Reviews and Meta-Analyses of Observational Studies (COSMOS-E) guidelines will be adhered to. Searches of key databases will be undertaken, and a two-phase screening process carried out by pairs of independent reviewers to select studies that meet the inclusion criteria. Data will be extracted using a purposefully designed form. Risk of bias will be assessed using the Joanna Briggs Institute Critical Appraisal Tools. If it is possible to pool prevalence data, a meta-analysis will be undertaken. Where pooling of data is not possible, a structured synthesis approach will be used, and results will be presented in tables and summarised narratively.

Conclusions

In recent years, there has been increased emphasis placed on promoting positive ageing and improving the healthcare experiences throughout the lifespan for people with neurodisabilities. Findings of this systematic review can inform decision-making related to healthcare for this vulnerable population and has the potential to contribute to reducing preventable deaths and premature mortality and promoting positive and healthy ageing for this group.

## Introduction

### Background

Neurodisability is an umbrella term for a range of lifelong physical and neurological conditions that are evident early in childhood, attributed to impairments of the brain or neuromuscular system and associated with functional limitations and difficulties with movement, cognition, hearing and vision, communication, emotion, and behaviour ^1^. Examples of diagnoses that fall under this umbrella include intellectual disability, Down syndrome, cerebral palsy, spina bifida and autism spectrum disorder ^2^. Improvements in life expectancy mean that the majority of people with neurodisabilities are now surviving well into adulthood ^3, 4^. This is a result of improvements in medical care and positive changes in societal attitudes towards, and treatment of, people with disabilities. It has implications for healthcare and disability service providers, however, with many struggling to meet the needs of this group. Adults with neurodisabilities, particularly those with more severe and complex disability, report difficulties finding adult healthcare providers that are specialised and familiar with the complexities of childhood-onset disabilities ^5, 6^.

### Common health issues for adults with neurodisabilities

In addition to experiencing complications related to their underlying diagnosis, adults with neurodisabilities are at a greater risk of developing many other ageing-related health conditions compared to the general population ^7, 8^. For this population, ageing-related conditions frequently differ from those encountered in the general population in terms of how they manifest, how frequently they present, the age of which they first arise, and the rate at which they progress ^9^. In addition, there are high levels of multi-morbidity, particularly among those with more severe levels of disability ^8, 10^.

Among the most common health problems for adults with neurodisabilities are disorders of vision and hearing; dental disease; cardiovascular disease; gastrointestinal and feeding problems; endocrine and metabolic diseases, including diabetes and thyroid disease; musculoskeletal disease, including osteoporosis; disorders of mental health, including anxiety and depression; and respiratory disorders, which are among the most common causes of death in this group ^7, 9, 11– 13^. Adults with intellectual disability, particularly those with Down syndrome, also have an increased risk of dementia, with data from Ireland showing the risk of developing dementia in people with Down Syndrome to be 88% by the age of 65, significantly higher than in the general population ^8^. Problems related to congenital heart anomalies and their sequelae also need to be considered among adults with Down syndrome ^14, 15^.

For adults with cerebral palsy, changes to mobility and general gross motor skills over time are also a concern. Among those who are independently ambulant during childhood, deterioration in walking skills and overall mobility can occur in adulthood, typically around the age of 35 ^16^. This can be closely associated with other symptoms such as pain, which is reported by 65–70% of adults with cerebral palsy ^17, 18^. Other problems frequently reported by this group include changes in the severity of the movement disorder, chronic fatigue, and increased risk of stroke ^19^.

### Equity of access to healthcare and unmet health care needs

While life expectancy for people with neurodisabilities has increased in most Western countries over the last few decades, it is still lower than that of the general population. In Ireland, for example, people with an intellectual disability are dying, on average, twenty years earlier than the general population ^3, 20^. Similar figures have been reported in Australia ^11, 21^, England ^22^, and Canada ^23^. This decreased life expectancy is often attributed to multi-morbidity and the medical issues that occur more frequently and earlier in life compared to the general population. In recent years, however, concern has been expressed about excess premature mortality and preventable deaths in this group ^11, 24– 26^.

Preventable deaths are those that could potentially be avoided by optimal health care and public health interventions that focus on the broader determinants of health, including behavioural and lifestyle factors, socioeconomic status, and environmental factors ^27^. Therefore, premature mortality and preventable deaths among adults with neurodisabilities highlight the importance of exploring equity of access to healthcare and the effectiveness of public health interventions for this group. It is well-documented that adults with disabilities face many barriers when it comes to accessing healthcare, including problems finding suitable healthcare providers, difficulties navigating complex healthcare systems, and issues related to communicating with healthcare providers ^28, 29^. They are also less likely than the general population to participate in cancer screening programmes ^30, 31^, public health interventions, and health promotion activities ^32^.

Unmet healthcare needs are a key indicator of equitable access in a quality healthcare system ^33^. Defined as *“the difference between the health care services deemed necessary to deal with a particular health problem and the actual services received”* ^34^, unmet healthcare needs can arise as a result of features of a health system or as a result of individuals’ circumstances ^35^. The latest combined data from two European surveys - the European Union Statistics on Income and Living Conditions (EU-SILC) instrument and the second wave of the European Health Interview Survey (EHIS), indicate that across the 28 EU countries surveyed, 26.5% of the population have at least one unmet healthcare need due to costs, distance or waiting lists ^36^. A limitation of this data, however, is that it does not give specific information on unmet healthcare needs for vulnerable groups, including adults with disabilities. In general population studies, being female, younger in age, and having high healthcare needs are reported to be associated with having a greater number of unmet healthcare needs ^37^. It is unclear if these patterns are replicated in adults with neurodisabilities.

### Rationale for this systematic review

There is a gap in the literature in relation to a synthesis of the evidence around unmet healthcare needs for adults with neurodisabilities. As a useful indicator of equity in access to healthcare, it is important to understand the prevalence and types of unmet healthcare needs in this group, and the factors associated with these unmet needs. As the number of people living for longer with neurodisabilities increases, and as disability advocates strive for a ‘lifecourse’ approach to healthcare for this group ^38, 39^, the findings of this review will provide evidence to inform service decision-making related to this population. In this review, particular attention will be given to the association between age and unmet healthcare needs.

### Objectives of this systematic review

This systematic review will address the following objectives:

1. To estimate the prevalence of unmet healthcare needs in adults with neurodisabilities.
2. To identify the types of unmet healthcare needs most frequently experienced by adults with neurodisabilities.
3. To explore the reasons for unmet healthcare needs (e.g. travel, wait-lists) and the factors associated with unmet healthcare needs (e.g. age, diagnoses) in adults with neurodisabilities.
4. To summarise the overall quality of the available literature relating to unmet healthcare needs in adults with neurodisabilities.

## Protocol

The systematic review will adhere to the Conducting Systematic Reviews and Meta-Analyses of Observational Studies (COSMOS-E) guidelines ^40^. We will also follow the Preferred Reporting Items for Systematic Reviews and Meta-Analyses (PRISMA) standardised reporting guidelines to standardise the conduct and reporting of this research ^41^. This protocol has been submitted for registration on PROSPERO international prospective register of systematic reviews (registration number 247805).

### Study eligibility criteria

***Study design.*** Observational studies, including cohort, case control, cross sectional, ecological studies, and other studies that include surveys as a means of collecting data relating to unmet healthcare needs, will be included. Intervention studies that include measures of unmet healthcare needs in the baseline assessments will also be included. Qualitative studies, review articles, dissertations, editorials, commentaries, and conference abstracts, will be excluded. Mixed methods studies will be included if the data from the quantitative element of the study can be extracted.

***Population.*** The study population of interest is adults aged 18 years and over with one of the following diagnoses: intellectual disability, cerebral palsy, autism spectrum disorder, Down syndrome, Rett syndrome, spina bifida, or Prader-Willi syndrome. We have included these diagnostic categories as they are reported to be the most prevalent childhood-onset neurodisabilities, and frequently co-occur ^2^. Studies that include both children and adults will be included if they report the results separately for participants aged 18 years and over. Studies will be excluded if they include participants with other diagnoses unless they report results separately for participants that have one of the fore-mentioned diagnoses. Given that the views of individuals with neurodisabilities are under-represented in the literature, studies that include patient, proxy, or provider reports of unmet healthcare needs in adults with neurodisabilities will be included.

***Outcomes.*** The outcome of interest is unmet healthcare needs, or more specifically: (i) prevalence of unmet healthcare needs; (ii) types of unmet healthcare needs; (iii) reasons for unmet healthcare needs; (iv) factors associated with unmet healthcare needs (age, gender etc.). For this systematic review, healthcare includes medical, nursing, dental, and allied health services, and unmet healthcare needs are considered to be situations where a person did not receive the healthcare that they needed at the time required. Studies that focus on other unmet needs will be excluded if they do not report the results for unmet healthcare needs separately.

### Search strategy

A comprehensive search strategy has been developed by the authors, in collaboration with a dedicated Education and Health Sciences librarian at the University of Limerick. The following databases will be searched for primary research studies, published in the English language, from inception to the date of the search: Cumulative Index of Nursing and Allied Health Complete (CINAHL), EMBASE, PubMed and EBSCO MEDLINE. Search terms will be based on the following keywords: unmet healthcare needs, neurodisability, and access to health care. All search terms and keywords will be combined with the relevant medical subject headings for each of the databases. Search terms will be combined using Boolean operators as appropriate. An example of the search strategy for PubMed is outlined in Table 1. The reference lists of included studies will also be searched.

**Table 1.** Sample PubMed Search Strategy.

#1	unmet healthcare need OR unmet health care need OR unmet need OR unmet psychiatric need OR unmet physical need OR access to care OR access to healthcare OR healthcare services access OR health services access OR health services availability OR healthcare demand OR treatment barriers OR health disparit* OR needs assessment **[Title/Abstract]**
#2	Needs Assessment OR Health Services Accessibility OR Patient Acceptance of Health Care OR Healthcare disparities OR Health Services Needs and Demand **[MeSH Terms]**
#3	#1 OR #2
#4	neurodisabilit* OR developmental disabilit* OR intellectual disabilit* OR intellectual impairment OR learning disability OR learning disorder OR cognitive impairment OR intellectual deficiency OR special needs OR mental retardation OR mentally disabled persons OR fragile X syndrome OR cerebral palsy OR down syndrome OR downs syndrome OR Rett syndrome OR spina bifida OR prader willi OR autism OR asperger syndrome OR childhood disintegrative disorder OR heller’s syndrome OR pervasive developmental disorder **[Title/Abstract]**
#5	Developmental Disabilities OR Developmental Disability Nursing OR Cerebral palsy OR Intellectual disability OR Persons with Mental Disabilities OR Cognitive Dysfunction OR Down syndrome OR Autistic disorder OR Autism Spectrum Disorder OR Rett syndrome OR Spinal Dysraphism OR Spina Bifida Cystica OR Spina Bifida Occulta OR Prader-Willi Syndrome **[MeSH Terms]**
#6	#4 OR #5
#7	#3 AND #6

### Screening

All titles and abstracts retrieved through the searches will be downloaded to an Endnote database and duplicates will be removed before a two-phase screening process is undertaken. During the first phase, all titles and abstracts will be screened, while in the second phase, the full-texts of all the retained articles will be screened against the inclusion criteria to confirm eligibility for inclusion in the final review. Screening will be performed by pairs of reviewers working independently (EM, AG, AW) and disagreements about inclusion will be resolved by another reviewer (KR, CK). Reasons for exclusion of articles in the second phase of screening will be identified and recorded. A PRISMA flow diagram will be used to illustrate the study selection process.

### Data extraction

A data extraction form will be designed a priori and piloted by each of the reviewers. Data will be extracted by pairs of reviewers working independently (EM, JR, ROS), and any disagreements in data extraction will be resolved by another reviewer (RG, MM). The data extraction form will collect the following information:

### Study characteristics and methods

■ Authors
■ Year of publication
■ Location and setting
■ Study design
■ Method of data collection
■ Objectives
■ Inclusion and exclusion criteria
■ Start and end dates (if relevant)
■ Duration of participation (if relevant)
■ Definition of outcome (unmet health care needs)
■ Respondent
■ Outcome measures used

### Participants

■ Number of participants
■ Age
■ Sex
■ Types(s) of developmental disability
■ Other relevant participant characteristics

### Outcomes

■ Prevalence of unmet health care needs (expressed as a %) (objective 1)
■ Types of unmet healthcare needs (objective 2)
■ Reasons for unmet healthcare needs (objective 3)
■ Factors associated with unmet healthcare needs (objective 3)

Attempts will be made to contact study authors to obtain missing data when possible. All missing data will be noted in the data extraction form.

### Assessment of risk of bias

The risk of bias in each study will be assessed using the Joanna Briggs Institute Critical Appraisal Tools ^42^. For each included study, pairs of reviewers working independently will use the relevant checklist to appraise the evidence. If the reviewers disagree and cannot resolve their differences through discussion, a third reviewer will be consulted. Studies will not be excluded based on their risk of bias assessment.

### Data synthesis and analysis

If it is possible to pool results from studies reporting on prevalence of unmet healthcare needs, a meta-analysis will be performed (objective 1). Due to the likely heterogeneity between population and outcome variables, a random-effects model will be used ^43^, and statistical heterogeneity will be assessed using the I ^2^ statistic ^44^. We will assess low heterogeneity as an I ^2^ value of between 0% and 30%, medium heterogeneity as 31% to 50%, and high heterogeneity as above 50% ^44^. Sub-group analysis (e.g. by age, type of disability) will be carried out if possible. If statistical pooling of prevalence data is not possible, results will be presented in tables and summarised narratively. In relation to the other outcomes, there is likely to be significant heterogeneity between studies in terms of participants’ diagnoses, settings, and outcome measures, so a ‘structured synthesis’ approach will be used. Data will be grouped based on the category that best explains the heterogeneity between studies (e.g. by age, diagnosis, or outcomes). Data will then be presented in tables and the results summarised narratively.

## Discussion

This systematic review will synthesise existing evidence related to unmet healthcare needs in adults with neurodisabilities. It will explore factors associated with variation in the prevalence and types of unmet healthcare needs in this population.

### Strengths and limitations

Strengths of this systematic review include the methodological approach taken and the use of the COSMOS-E guidelines to inform its design. The detailed search strategy will ensure that a wide range of studies of populations with different diagnoses are located and will enable a thorough exploration of unmet healthcare needs during adulthood for this population. There are also a number of potential limitations. It is likely that there will be significant heterogeneity between studies, in terms of sample characteristics and outcome measures used. If this is the case, it may not be possible to combine data in a meaningful way or perform a meta-analysis. It is also possible that due to differences in terminology and the use of multiple definitions in the literature for both the exposure (‘neurodisability’) and outcome of interest (‘unmet healthcare needs’) in this review, there may be difficulties determining whether some studies should be included or not. The exposure and outcome of interest have been clearly defined in the methods section of this protocol in an attempt to prevent such situations arising, and plans are in place to consult with additional reviewers when conflicts arise. Because, we have specified in the inclusion criteria the specific diagnoses that will be included, studies of people with other types of neurodisability (e.g. neuromuscular disorders and certain rarer degenerative conditions; and specific learning disabilities such as developmental coordination disorder) will be excluded. Finally, the exclusion of studies published in languages other than English means that it is possible that important studies will be missed.

## Conclusions

Traditionally, research in neurodisability has focussed on improving outcomes and healthcare provision for children and adolescents; and there is less focus on the healthcare experiences and outcomes of this population during adulthood. In order to reduce healthcare inequities and health disparities and promote healthy ageing for adults with neurodisabilities, we need to understand more about the healthcare experiences of this group. Unmet healthcare needs are an important indicator of overall access to healthcare, and the findings of this systematic review may inform policy responses to ageing with disability.

## Study status

The study has not commenced yet. The searches will be undertaken, followed by screening, in June 2021.

## Data availability

No data are associated with this article.
