# Supplementary material for: Unmet healthcare needs in adults with childhood-onset neurodisabilities: a protocol for a systematic review
Source: HRB Open Res. 2021 Sep 28;4:107. [Version 1] doi: 10.12688/hrbopenres.13309.1 (PMC9399657; doi:10.12688/hrbopenres.13309.1)
Supplement: Peer review history

# Peer review: Reviewer response for version 1

**DOI:** 10.21956/hrbopenres.14489.r32654

**Published:** 2022-08-23

**Authors:** Shauna Kingsnorth

**Affiliations:** 1 Evidence to Care, Holland Bloorview Kids Rehabilitation Hospital, Toronto, ON, Canada; 2 Bloorview Research Institute, Toronto, ON, Canada; 3 Department of Occupational Science & Occupational Therapy, University of Toronto, Toronto, ON, Canada; 4 Rehabilitation Sciences Institute, University of Toronto, Toronto, ON, Canada

Thank you for the opportunity to review and comment. This article presents a protocol for a systematic review to capture the prevalence and identify types and reasons for unmet health needs in adults with neurodisabilities. Findings will be particularly important for both adult care planning as well as transition to adult services for youth and young people with childhood-onset disabilities.

 The protocol is very well structured with the use of recognized best practices. 

 A few minor recommendations for the team to consider: 

- Search may yield high number of hits. Consider being prepared for a calibration exercise as part of initial screening efforts to ensure reviewers are consistently applying inclusion criteria.
- Screening tool software or mobile apps such as Covidence or Rayyan may also be worth exploring following deduplication efforts. ^1^ 
- Expectation of heterogeneity is high. Objectives 2 and 3 will likely entail a mapping exercise to organize findings and/or describe patterns and/or consider explanatory factors as part of the narrative synthesis. Recommend boosting description of this 'structured synthesis' and potential tools/techniques that may be used (i.e., textual descriptions, groupings/clusters, tabulation, content, or thematic analysis).
- As outlined by Borges Migliavaca *et al.*'s paper (BMC Medical Research Methodology, 2020) ^2^ of a systematic review of systematic reviews of prevalence, it would also be prudent to acknowledge in the limitations that unpublished sources are out of scope and as such, sources such as clinical registries, census data, etc. will not be captured. The team's selection of the JBI Critical Appraisal Tool is consistent with this paper as well. 
- Could the authors' comment on their position on use of a formal quality assessment in the review? And implications for using/not using as part of methodology re: limitations or strengths.
- Is there a prior plan for dissemination and/or patient/clinical partner involvement in the study?

Is the study design appropriate for the research question?

Yes

Is the rationale for, and objectives of, the study clearly described?

Yes

Are sufficient details of the methods provided to allow replication by others?

Yes

Are the datasets clearly presented in a useable and accessible format?

Not applicable

Reviewer Expertise:

Childhood-onset disabilities, transition to adult services, synthesis methodologies

I confirm that I have read this submission and believe that I have an appropriate level of expertise to confirm that it is of an acceptable scientific standard.

## References

[1] : De-duplication of database search results for systematic reviews in EndNote. *J Med Libr Assoc* .2016;104(3) : 10.3163/1536-5050.104.3.014 240-3 10.3163/1536-5050.104.3.014 27366130PMC4915647

[2] : How are systematic reviews of prevalence conducted? A methodological study. *BMC Medical Research Methodology* .2020;20(1) : 10.1186/s12874-020-00975-3 10.1186/s12874-020-00975-3 32336279PMC7184711

# Peer review: Reviewer response for version 1

**DOI:** 10.21956/hrbopenres.14489.r30493

**Published:** 2021-10-26

**Authors:** Elisabet Rodby-Bousquet

**Affiliations:** 1 Department of Clinical Sciences, Orthopaedics, Lund University, Lund, Sweden

This is a clear and well-written study protocol and the topic is highly relevant. It may be a bit challenging to cover such a wide spectrum of diagnoses and outcomes but it is important to identify the gaps in health care. I would recommend the authors also include AMED the Allied and Complementary Medicine Database in their search.

 In several countries, pediatric and geriatric care include multi-professional teams, but for adults with childhood-onset neurodisabilities the regular health care system is usually fragmented and not well adapted for people with lifelong conditions. This is even more challenging for this population as they are at higher risk of fatigue, pain, decline in function from an early age and also to develop several diseases such as cardiovascular, respiratory, metabolic and mental health problems. Therefore, continuity of care is important through the lifecourse for people with more complex needs. This systematic review will increase our understanding of unmet health care needs in adults with childhood-onset neurodisabilities and I sincerely hope this will guide future interventions and actions to improve the health care system to better meet their needs within all areas.

Is the study design appropriate for the research question?

Yes

Is the rationale for, and objectives of, the study clearly described?

Yes

Are sufficient details of the methods provided to allow replication by others?

Yes

Are the datasets clearly presented in a useable and accessible format?

Yes

Reviewer Expertise:

Individuals with childhood onset disabilities through their lifespan, with special focus on cerebral palsy.

I confirm that I have read this submission and believe that I have an appropriate level of expertise to confirm that it is of an acceptable scientific standard.
